# Improved Photo-Ignition of Carbon Nanotubes/Ferrocene Using a Lipophilic Porphyrin under White Power LED Irradiation

**DOI:** 10.3390/ma11010127

**Published:** 2018-01-13

**Authors:** Paolo Visconti, Patrizio Primiceri, Roberto de Fazio, Antonio Paolo Carlucci, Selma Elaine Mazzetto, Giuseppe Mele

**Affiliations:** 1 Department of Innovation Engineering, University of Salento, Lecce 73100, Italy; patrizio.primiceri@unisalento.it (P.P.); defazio.roberto@studenti.unisalento.it (R.d.F.); paolo.carlucci@unisalento.it (A.P.C.); giuseppe.mele@unisalento.it (G.M.); 2 Laboratório de Produtos e Tecnologia em Processos (LPT), Departamento de Química Orgânica e Inorgânica, Universidade Federal do Ceará, Fortaleza 60440-900, Brazil; selma@ufc.br

**Keywords:** multiwalled carbon nanotubes, ferrocene, metal nanoparticle ignitors, LED source, photo-induced ignition, porphyrin

## Abstract

The aim of this work is to investigate and characterize the photo-ignition process of dry multi-walled carbon nanotubes (MWCNTs) mixed with ferrocene (FeCp_2_) powder, using an LED (light-emitting diode) as the light source, a combination that has never been used, to the best of our knowledge. The ignition process was improved by adding a lipophilic porphyrin (H_2_Pp) in powder to the MWCNTs/FeCp_2_ mixtures—thus, a lower ignition threshold was obtained. The ignition tests were carried out by employing a continuous emission and a pulsed white LED in two test campaigns. In the first, two MWCNT typologies, high purity (HP) and industrial grade (IG), were used without porphyrin, obtaining, for both, similar ignition thresholds. Furthermore, comparing ignition thresholds obtained with the LED source with those previously obtained with a Xenon (Xe) lamp, a significant reduction was observed. In the second test campaign, ignition tests were carried out by means of a properly driven and controlled pulsed XHP70 LED source. The minimum ignition energy (MIE) of IG-MWCNTs/FeCp_2_ samples was determined by varying the duration of the light pulse. Experimental results show that ignition is obtained with a pulse duration of 110 ms and a MIE density of 266 mJ/cm^2^. The significant reduction of the MIE value (10–40%), observed when H_2_Pp in powder form was added to the MWCNTs/FeCp_2_ mixtures, was ascribed to the improved photoexcitation and charge transfer properties of the lipophilic porphyrin molecules.

## 1. Introduction

Carbon nanotubes (CNTs), since their discovery, have attracted the attention of researchers and companies for their mechanical, electrical, thermal, and optical unconventional properties, and for their particular dimensions and chemical structure. Specifically, the CNTs photo-ignition process was observed, accidentally, by exposing single-walled carbon nanotubes (SWCNTs) to the light pulse of an ordinary camera [1]. The structural changes observed in the post-ignited samples in Ajayan et al. [2] suggested that the ignition was due to a local temperature increase (higher than 1500 °C) sufficient to initiate the carbon oxidation. For researchers, the local thermal increase is due to the high efficiency of CNTs in absorbing light radiation, because of their black color and their higher thermal conductivity with respect to the metallic nanoparticles. Thus, the heat confinement in nanostructures leads to drastic structural deformations and, under oxidant environments, induces the ignition process [2]. Smits et al. highlighted the importance of a metallic catalyzer, contained in SWCNTs, for the photo-ignition process [3]; in fact, when exposing samples of high-purity, as-produced SWCNTs (purity 99.5%, with iron (Fe) nanoparticles of 6–10 μm) to a camera flash, no reaction was observed. On the contrary, by using HiPCo SWCNTs (high-pressure carbon monoxide SWCNTs) produced by Carbon Nanotechnologies, Inc., containing about 30 wt % of metal impurities or Fe powder, photo-ignition was induced. Moreover, by employing TEM (transmission electron microscopy) methods, the authors analyzed samples of the three nanomaterials (high-purity SWCNTs, HiPCo SWCNTs, and Fe powder) after flash exposure, highlighting that heat is locally confined in Fe nanoparticles rather than a SWCNTs bundle, which can thus gain enough thermal energy to oxidize [3]. Results reported in [3] for SWCNTs have also been confirmed for MWCNTs in [4]. Further qualitative descriptions of the photo-ignition process, determining the roles and functionalities of the different stage of the combustion, were given in [5].

A chemical-physical interpretation of the MWCNTs/FeCp_2_ (MWCNTs/ferrocene) photo-ignition process is reported in our previous research work [6]: photon absorption induces the FeCp_2_ photo-excitation, resulting in electron transfer processes from FeCp_2_ to MWCNTs. The obtained radical species (FeCp_2_^+^ and MWCNTs^−^) can react with oxygen, triggering combustion. Moreover, we highlighted the dependence of the photo-ignition process on wavelength range and luminous power.

Braidy et al. [7] focused their study on the analysis of SWCNT samples after photo-ignition; by means of X-ray diffraction, the authors observed the presence of iron oxide particles in the combustion byproducts, mainly Fe_2_O_3_ (iron(III) oxide) with traces of Fe_3_O_4_ (iron(II,III) oxide). Furthermore, by performing a TEM investigation, two different morphologies of oxide particles were observed: smaller nanoparticles inside a network of SWCNTs residuals and bigger particles randomly interconnected or fused in grain. The large increase in Fe particle size suggested that, inside the SWCNTs network, temperatures higher than that of iron melting (1538 °C) were reached, thus confirming the hypothesis in [1]. Also, Tseng et al. showed that the amount of Fe catalyst embedded in CNTs plays a key role in ignition, indicating that a higher content of catalyst in the mixture ensures easier ignition [8]. Also other nanomaterial typologies were employed as ignitor agents; for example, in [9], the authors demonstrated that aluminum nanoparticles (Al NPs) can be ignited by a camera flash through the photothermal effect.

A quantitative analysis of the photo-ignition process of as-produced SWCNTs (with 50% Fe content), with varying pulse duration and wavelength ranges, was reported in [10,11,12,13]; the results highlighted that, regardless of the wavelength ranges of the incident light beam, the MIE (minimum ignition energy) values depend on the light pulse duration. In particular, a 30–35 mJ/pulse was required for igniting an uncompacted sample in steady air with a light pulse duration of 0.1 ms, whereas the 80–90 mJ/pulse had a pulse duration of 9 ms [10]. In addition, the authors studied the dependence of MIE values on Fe concentration using light pulses of 9 ms; the results confirmed that increasing Fe concentration caused a decrease in MIE values. Similar characterizations relative to the photo-ignition of dry MWCNTs/FeCp_2_ mixtures were performed in [14,15,16,17]; in particular, the authors investigated the dependence of MIE values on the concentration by weight of ferrocene inside the sample. Obtained results indicated that a higher ferrocene amount involves a lower luminous energy to initiate sample combustion; these results were unexpected and different from those previously reported in the literature [10,11,12,13], where, however, the photo-sensitive nanomaterials used were SWCNTs and Fe nanoparticles instead of the MWCNTs and ferrocene employed in [14,15,16,17].

The application of CNTs’ photo-ignition is mainly in the combustion and propulsion sectors, where photo-ignition properties are used for triggering the combustion of solid, liquid, and gaseous fuels, thus allowing us to improve the combustion features [16,17,18,19,20,21,22,23]. In [21], the authors patented the use of a nanostructured material for obtaining distributed ignition and combustion improvements in propulsion applications, such as HCCI (homogeneous charge compression ignition) engines, liquid rocket fuel, and enhanced flame stabilization in gas turbines. 

In [22], the authors have demonstrated for the first time the promotion of flame acceleration and deflagration-to-detonation transition (DDT) using distributed photo-ignition of SWCNTs suspended in fuel/oxidizer mixtures. Numerous research works focus on the analysis of fuels’ combustion features induced by photo-igniting nanomaterials [16,17,18,19,23,24].

Although the CNTs photo-ignition phenomenon was observed for the first time by using a Xenon (Xe) lamp, which, until now, has been the light source most often used for photo-igniting nanomaterials; however, its usage has numerous limitations. In fact, an Xe lamp requires both high supply voltages and high trigger signals and, in addition, has intrinsic mechanical instability and a reduced lifetime. Furthermore, existing circuital solutions do not allow for running the Xe lamp at frequencies higher than 40 Hz. For all these reasons, when looking for alternative ignition sources, a white power LED source has been considered the optimal choice; in fact, given the rapid development of the semiconductor industry over the last years, white power LEDs have acquired the potential to represent a suitable alternative to the Xe lamp, both for luminous power and frequency spectrum. In particular, LEDs feature higher luminous efficiency (higher than 150 lumen/W) than the Xe lamp (40–50 lumen/W), resulting in lower power consumption, higher mechanical stability, and a longer lifetime (more than 100,000 h). Finally, a pulsed LED source can be easily powered and controlled, allowing it to reach frequencies much higher than those obtainable from a Xe lamp and allowing for the easy adjustment of the emitted luminous intensity.

This research work focuses on the analysis and characterization of the dry MWCNTs/FeCp_2_ ignition process using LED sources for triggering sample combustion. In a first test campaign, ignition tests of MWCNTs/FeCp_2_ samples using a continuous emission LED source were performed; in a second test campaign, using a proper light source composed of four LEDs and a properly designed and realized driving electronic unit, ignition tests using a single luminous pulse were carried out. For improving the ignition mechanism, porphyrin powder, i.e., H_2_Pp porphyrin, was added to the MWCNTs/FeCp_2_ mixtures. Porphyrins are considered very attractive photo- and radio-sensitizers that can find extensive application in chemical technology, ecology, medicine, and electronics. Some composites containing porphyrin derivatives allow a rapid charge separation and slow recombination, key factors for obtaining high light conversion efficiencies [25,26]. In recent years, many efforts have been made towards the development of novel porphyrins to enhance electron transfer efficiency to semiconductors, besides the possibility of using porphyrins derived from natural and renewable sources (such as the cardanol present in the cashew nut shell liquid) in order to make the photocatalytic process even more environmentally friendly [27]. In this research work, the lipophilic porphyrin used presents absorption peaks very close to the emission spectrum peaks of the employed LED source. The obtained experimental results show that the porphyrin powder, added to the MWCNTs/FeCp_2_ mixture, allows us to improve the photo-ignition process, resulting in MIE values about 10–40% lower than those needed to ignite samples without porphyrin.

## 2. Materials and Methods 

In this section the description of preparation modes of MWCNTs/FeCp_2_ samples, in addition to the experimental setup and operating modalities for performing photo-ignition tests by means of a continuous-emission LED source and a single luminous pulse, are reported. Finally, improvements in the ignition mechanism of MWCNTs/FeCp_2_ mixtures by adding porphyrin powder are analyzed.

### 2.1. Sample Preparation

In this research work, two different typologies of MWCNTs with different chemical and physical features were tested; the first typology, named high-purity MWCNTs (HP-MWCNTs), is produced by SouthWest NanoTechnologies, Inc. (Norman, OK, USA), featuring an external diameter of 10 ± 1 nm, length of 3–6 µm and purity higher than 98 wt %. The second typology, Industrial Grade MWCNTs (IG-MWCNTs), is produced by Nano Lab Inc. (Waltham, MA, USA) and features an external diameter of 10–30 nm, length of 5–20 µm, and purity higher than 85 wt %, with residual impurities of iron, ceramic, and oxides. In the following, the preparation mode of MWCNTs/FeCp_2_ samples and the weight ratios reported in Table 1 are described: after weighing the two components (both MWCNTs and ferrocene in powder form) according to the desired weight ratio, the two powders were manually mixed using a ceramic mortar, thereby obtaining a mixture with no evident agglomerations, as observed by the optical microscope (45X 6W LED Trinocular Microscope, AmScope, Irvine, CA, USA).

#### 2.1.1. Experimental Setup for Ignition Tests Using Continuous-Emission LED 

**Figure 1:**
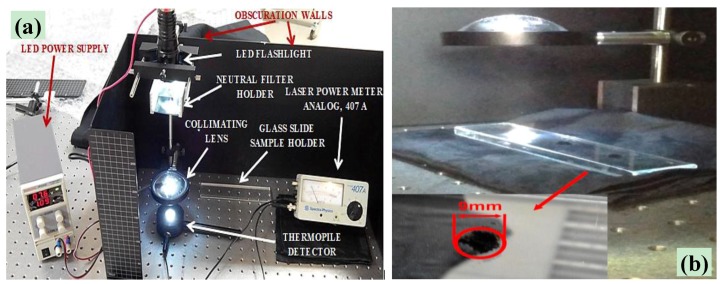
Setup used for photo-igniting the dry MWCNTs/FeCp_2_ samples by using the continuous emission XM-L T6 LED (**a**) and view of the three samples with diameter of 9 mm, placed on a quartz plane, sequentially exposed to LED source (**b**).

The experimental setup employed for performing photo-ignition tests, by means of a continuous-emission LED source, is shown in Figure 1a; the LED-based source used integrates a white high-power LED (XM-L T6, Cree Inc., Durham, NC, USA). As shown in Figure 1a, the experimental area used for experimental tests was laterally delimited by metallic walls and covered by a black cloth to avoiding a sample’s exposure to ambient light, which could change the sample’s state.

In front of the LED source was placed a support for mounting neutral optical filters, used for changing LED optical intensity and thus determining the minimum power for the photo-ignition of MWCNTs/FeCp_2_ samples. For collimating the luminous beam, so confining most of the emitted luminous power on a small area, a collimating lens with a diameter of 3 inches and a focal length of 10 cm was used; it was placed in front of the light source, as indicated in Figure 1a, in order to gather most of the light emitted from the LED. In this way, at sample height, a square area of 0.64 cm^2^ (8 mm × 8 mm) was obtained. For measuring the luminous intensity that reaches samples, the Power Meter Analog 407 shown in Figure 1a was employed; it uses a thermopile for measuring luminous intensity for a range up to 20 W, allowing us to detect luminous power in the UV–IR range, without losing accuracy in response (absorbance changes by 1% in the 400–1000 nm range). Finally, the LED driver embedded in the torch was fed with 7.6 V voltage and the absorbed current was equal to 1.09 A; in this way, the luminous intensity of LED source, without any neutral filter, was measured (340 mW).

Three samples of the MWCNTs/FeCp_2_ mixture, with the same weight ratio, in the form of small heaps of weight 6.6 mg and diameter 9 mm, were placed on a quartz plane; hence, three independent tests were carried out each time. The quartz plane was placed on a black cloth for minimizing the light reflected by the metallic bench (Figure 1b). Subsequently, by placing the MWCNTs/ferrocene sample under the luminous spot, the triggering of combustion was shown by the emission of smoke and burnt particles from the sample. The combustion process was recorded using a high-resolution camera (Canon PowerShot SX60 HS, Canon, Tokyo, Japan) for extrapolating, after proper post-processing, its physical and temporal features. 

#### 2.1.2. Experimental Setup for Photo-Ignition Tests Using a Pulsed LED Source

**Figure 2:**
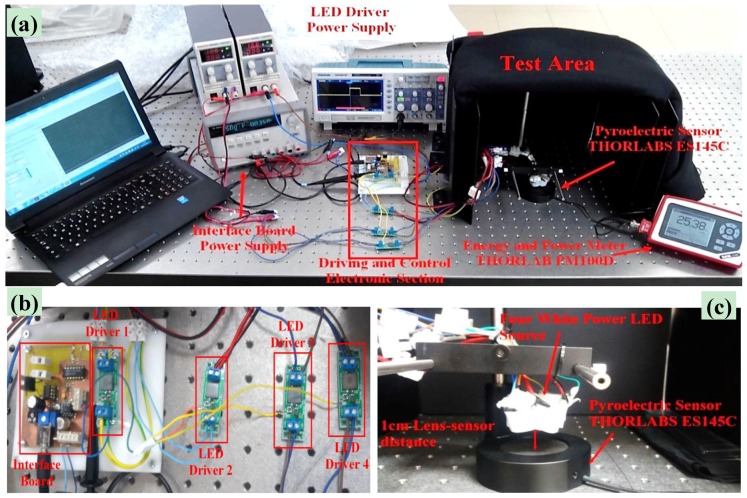
View of experimental setup used for performing ignition tests by means of pulsed LED source (**a**); the used four LED drivers and the PC-interfaced board (**b**); and the test area with four white power LEDs and the pyroelectric sensor (**c**).

In Figure 2a, the experimental setup used to perform the ignition tests by means of a pulsed LED source is shown. It is composed of two different sections: the electronic driving and control section, shown in Figure 2b, which includes an interface board (PC-interfaced) and four LED drivers, one for each used LED; and the test area, shown in Figure 2c, where the ignition tests of MWCNTs/FeCp_2_ samples were carried out. The PC-interfaced board was used for adapting the voltage level of the enabling signal, produced by means of a proper LabVIEW application with the desired duration on the audio channel of a PC, to the voltage range as required by the dimming/enabling input of the LED driver. In this way, the output signal of each LED driver, with the same duration of the enabling signal, turned on properly the white power LED. A digital oscilloscope (Hantek DSO5072P, Hantek, Qingdao, China) was used for verifying the duration of the signal provided to the LED driver and detecting the current instantly absorbed from the LED source (by using a sensing resistor in series to a white power LED).

**Figure 3:**
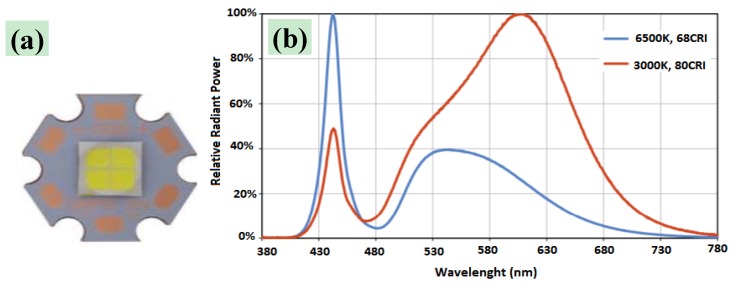
Top view of Cree XHP70 6500 K LED, 12 V version mounted on its proper 20 mm copper board (**a**) and emission spectra of Cree XHP70 models; those employed have color temperatures of 6500 K (blue trace) and 5000 K (not reported) (**b**).

The LEDs source, composed of four white power LEDs (model XHP70, Cree Inc., Durham, NC, USA), was fixed to a support anchored to the test bench. A collimation optic was applied on each LED; the resulting LED source was placed about 1 cm from the tested samples, the distance at which the emitted light was maximally collimated. Also, for these tests as well as for tests with a continuous-emission LED source, the test zone was laterally delimited by metallic walls and covered with a cloth for avoiding sample exposure to environmental light. The XHP70 LED used combines high luminous flux (maximum light output: 4200 lumen), luminous efficiency (150 lumen/W at binning conditions), robustness, and small dimensions (7 × 7 mm). This LED model has a dual configurable power supply depending on PCB layout: 6 V of nominal voltage with maximum current of 4.8 A or 12 V (used model in this work) with maximum current of 2.4 A. The 12 V version of the LEDs, mounted on a proper copper board, is shown in Figure 3a; moreover, two LEDs featuring color temperatures of 5000 K and two of 6500 K were used (Figure 3b).

**Figure 4:**
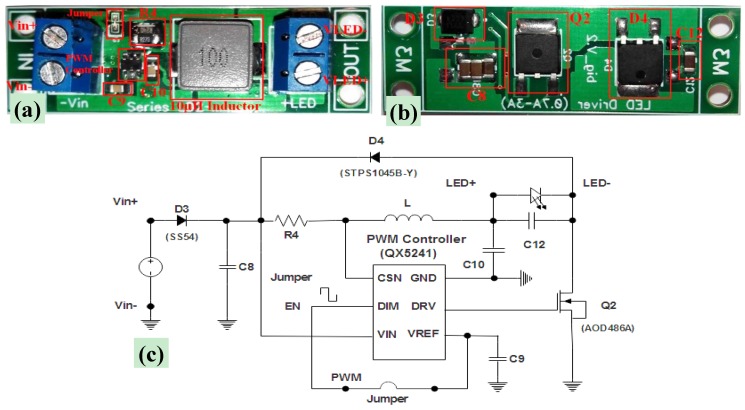
View of LED driver (constant current buck driver) with the different components highlighted: top view (**a**); bottom view (**b**); and related circuital scheme (**c**).

For driving the white power LEDs, drivers specially designed for Cree XHP70 LEDs (Figure 4a–c) were used; defined as constant current buck drivers, they employ a buck circuital topology and operate by providing a supply voltage regulation by means of a PWM controller, by adjusting the conversion duty cycle and/or switching frequency, operations also needed for setting the output current to a prefixed value. In fact, the LED driver uses a high-side (toward to the power-supply) sensing resistor for detecting the current that instantly flows through the inductor and thus the current absorbed by LED, allowing for highly accurate control (up to ±1%) of the output current; in addition, the value of this resistor sets the desired output current (in this case 2.4 A, i.e., the maximum operating current of the LED used).

The device has a dedicated dimming/enable input used, in our tests, for generating the single luminous pulse, as previously described. Finally, the LED driver features an input voltage range of 5–27 V, an output voltage range between 2 V and 14 V, and an output current of 2.4 A, with a nominal efficiency equal to 92%.

**Figure 5:**
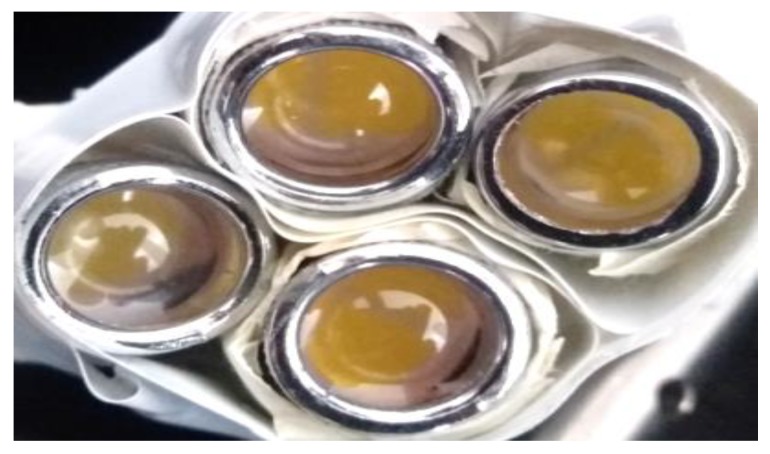
View of the four LEDs, with related optics, used for generating the single light pulse for the ignition tests; LEDs were slightly inclined in order to direct the light to a small area, thus increasing the luminous density.

Considering the minimum luminous energy values needed for igniting the MWCNTs/FeCp_2_ samples, obtained in our previous works [14,15,16,17], high luminous intensity values are required; for this reason, as cited above, the LED source is composed of four LEDs arranged together, as shown in Figure 5, for simultaneously collimating the emitted light on areas of the order of 1–2 cm^2^. Therefore, since each LED emits with an emission angle of 120°, a small optic consisting of a small tube with a length of 1.3 cm and a diameter of 0.8 cm and a small semi-convex lens with a focal length of 5 cm were applied on each LED.

**Figure 6:**
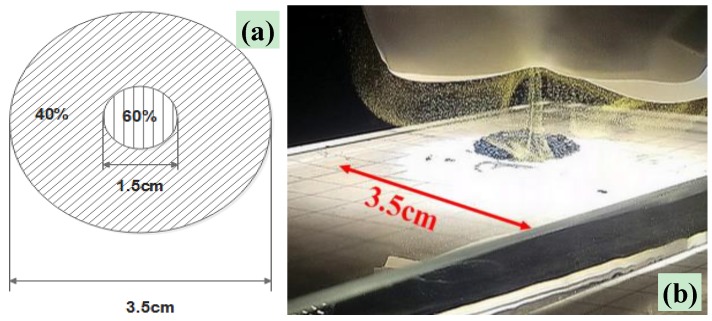
View of assumed light distribution emitted from the four-LED source for the single-pulse tests (**a**) and frame extrapolated from acquired video during the performed tests, with the luminous spot dimension highlighted (**b**).

Based on experimental observation, the emitted light was not uniformly distributed on a circular area with a diameter of 3.5 cm; therefore, it was assumed that the luminous intensity distribution, or equivalent pulse energy distribution, at 1 cm of lens–sensor distance, was equal to 60% in the circular area with a diameter of 1.5 cm and 40% in the remaining area, as shown in Figure 6a. This assumption was extrapolated by frames obtained from the high-resolution videos acquired during ignition tests (Figure 6b). Therefore, an effective illuminating area ($A_{eff}$), corresponding to a uniformly illuminated area, was calculated (formula below) as a weighted average according to the supposed energetic distribution:

(1) $$A_{eff}=p_{1}\cdot A_{1}+p_{2}\cdot A_{2}=0.6\times 1.76\;{\mathrm{cm}}^{2}+0.4\times 7.86\;{\mathrm{cm}}^{2}=4.2\;{\mathrm{cm}}^{2}$$

Assuming that the luminous energy was confined to a circular area, the radius of this circle is given by:

(2) $$r_{3}=\sqrt{\frac{A_{eff}}{\pi }}=\sqrt{\frac{4.2\;{\mathrm{cm}}^{2}}{\pi }}=1.15\;\mathrm{cm}\;$$

Therefore, we can assume that the luminous energy was uniformly distributed on a circular area with a diameter of 2.3 cm.

Before carrying out ignition tests, the luminous energy emitted from the LED source was measured as a function of pulse duration by employing the THORLABS PM100D optical power/energy meter (shown in Figure 2a), equipped with the THORLABS ES145C pyroelectric sensor, and by keeping the lens–sensor distance equal to 1 cm. The pyroelectric sensor used, as reported in its datasheet, is able to perform energy measurements of light pulses with a duration from sub-ns up to ms; therefore, for pulse durations higher than 2.5 ms, once luminous power for short pulse durations was calculated as reported in Equation 3, pulse energy values were linearly extrapolated.

(3) $$P=\frac{E_{pulse}}{\Delta t}=\frac{25.38\;\mathrm{mJ}}{2.5\;\mathrm{ms}}=10.15\;W$$

Since the LED source used emits a constant luminous intensity P [W] (assuming the absorbed current is constant), the energy E [J] of a light pulse with duration Δt [s] is given by:

(4) E=P·Δt

After characterization of the pulsed LED source, the MIE values for different IG-MWCNTs/FeCp_2_ weight ratios were obtained. For the single pulse ignition tests, three samples of weight 6.6 mg and 9 mm diameter, prepared according to the modalities described in the previous section, were placed, each time, on a quartz plane in the form of equally-spaced heaps. Ignition tests were performed starting with long pulse durations (200 ms) and, once the sample ignition was verified, they were progressively reduced until no combustion was observed. Between consecutive ignition tests, the IG-MWCNTs/FeCp_2_ sample was also substituted in case no ignition was observed. In addition, for better visibility of smoke and combustion particles emitted as a result of combustion, a test zone near the sample was illuminated by means of continuous-emission LED sources with low intensity. 

## 3. Results 

In this section, the experimental results obtained from the photo-ignition tests carried out on dry MWCNTs/FeCp_2_ samples, using continuous-emission white power LED and the two MWCNTs typologies, are reported; subsequently, results of ignition tests by means of a single light pulse employing the experimental setup described in the previous section are shown. Finally, an analysis and discussion of the obtained results related to the different ignition tests carried out, in addition to the results of photo-ignition of dry IG-MWCNTs/FeCp_2_ samples enriched with porphyrin, are presented.

### 3.1. Results of Ignition Tests Using the Continuous-Emission LED Source

**Figure 7:**
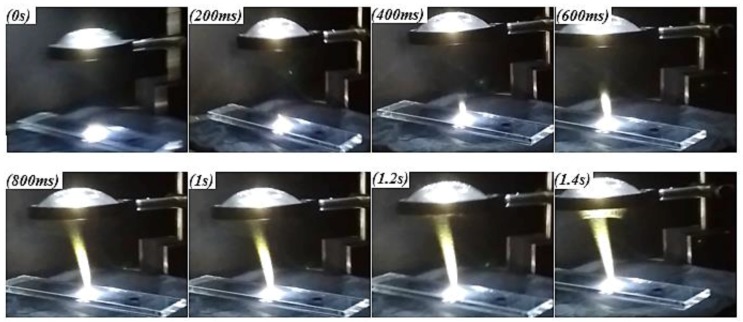
Frame sequence, 200 ms temporally spaced, extrapolated from video recorded during the ignition test of an HP-MWCNTs/FeCp_2_ sample with a 1:3 weight ratio.

For carrying out ignition tests, the HP-MWCNTs/FeCp_2_ nanoparticle typology was the first to be used; before the sample exposure, the luminous power emitted from LED source was measured by means of a Laser Power Meter Analog 407A (Newport Corporation, Irvine, CA, USA) without any neutral optical filter interposed between the LED and the pyroelectric sensor, but only the lens used for collimating light beam. Subsequently, once we acquired the luminous power and removed the sensor, the nanomaterial sample was positioned on the quartz plane at the same height as the sensor under the luminous spot; a high-resolution camera recorded the combustion process. Figure 7 gives the frame sequence of the ignition of a HP-MWCNTs/FeCp_2_ sample with a 1:3 weight ratio (luminous power = 340 mW); frames are temporally spaced at 200 ms.

The minimum luminous power needed for igniting the samples was determined by progressively reducing the luminous power on the sample by using neutral optical filters with higher optical density (OD). Each time, if sample ignition occurred, a new sample with the same weight ratio and a filter with higher OD than the previous one were used until no ignition was observed. Between one test and the next, the luminous power emitted from the LED was measured each time for each neutral filter used.

Since the resolution on luminous power measurements was limited by the OD values of the neutral optical filters, ignition thresholds were extrapolated based on experimental observation (ignition delay Δt and visual combustion intensity) of different ignition tests performed on samples with the same weight ratio. When sample ignition was observed after a time interval ∆*t* equal to 2 s and/or with low combustion intensity, the ignition threshold was assumed to be equal to the measured power corresponding to the neutral optical filter used. 

In Table 2, the power ignition thresholds obtained for each tested HP-MWCNTs:FeCp_2_ weight ratio (i.e., from 4:1 to 1:4), referring to an illumination area of 0.64 cm^2^, are reported, together with the relative power density and neutral optical filter used. As indicated in Table 2 and explained previously, for all tested samples’ weight ratios, except for the 3:1, the ignition threshold was assumed to be the luminous power value measured with the neutral optical filter with which the ignition was observed; instead, for a 3:1 weight ratio, the ignition threshold was extrapolated based on experimental observation related to ignition delay and ignition intensity, visually evaluated from the acquired high-resolution videos.

Table 2 reports a comparison between ignition thresholds obtained by using the continuous-emission LED source and those obtained using a CW Xe lamp. The ignition thresholds are shown in terms of both luminous power referring to an illumination area of 0.64 cm^2^ and power density. The threshold values obtained with the LED source are considerably lower than those obtained using the CW Xe lamp for all the tested weight ratios. In particular, the 1:3 weight ratio of HP-MWCNTs/FeCp_2_ samples presents the lowest ignition threshold. As previously reported, the ignition threshold decreases as much as the ferrocene amount in the sample increases when continuous-wave Xe was used as the irradiation lamp [6]. 

In this work two different light sources, i.e., LED-based combined with a Xe lamp, were used. Different threshold values have been ascribed to the different emission spectrum of the LED source compared with the Xe lamp. In fact, the absorption spectrum of the HP-MWCNTs/FeCp_2_ mixture shows that mixture absorption increases towards the ultraviolet (UV) region and has low values in the infrared (IR) region. Therefore, since the Xe lamp has a very wide emission spectrum, nearly uniform from UV to the deep IR region, the IR components are weakly absorbed by the nanomaterial and thus their contribution to the photo-ignition process is negligible. On the contrary, the LED source has a relevant emission spectrum only around the visible (VIS) and near-UV region; consequently, a higher portion of the emitted energy is absorbed by the sample, resulting in a lower value of luminous power for triggering the ignition. In other words, the higher values of ignition thresholds obtained with the Xe lamp are due to the fact that the thermopile sensor acquires luminous power in the whole wavelength range covered by the lamp; hence, for achieving the light power needed to ignite the samples, besides the luminous power related to the UV–VIS range, power related to IR region is also detected, resulting in higher luminous power values for obtaining ignition.

**Figure 8:**
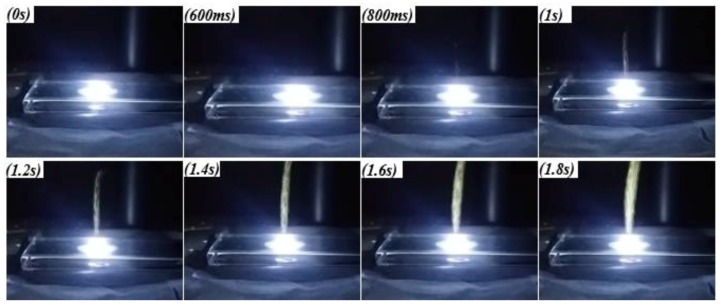
Frames sequence relative to the photo-ignition of a sample with a 1:3 weight ratio (IG-MWCNTs:ferrocene); frames are temporally spaced by 200 ms.

Once we completed the test campaign using the HP-MWCNTs nanoparticles, the other typology of MWCNTs (i.e., the industrial-grade MWCNTs), with dimensional and purity features different (worse) than those of the high-purity MWCNTs, was used in a new photo-ignition test campaign. Therefore, by using the experimental setup previously employed, with the same continuous-emission LED source, ignition thresholds were determined and compared to those obtained with dry HP-MWCNTs/FeCp_2_ mixtures for highlighting the eventual dependence of ignition thresholds on nanotubes’ features. The experimental procedure was the same as that followed for the HP-MWCNTs/FeCp_2_ mixtures described above; Figure 8 reports the frame sequence, temporally spaced by 200 ms, related to the ignition test of a IG-MWCNTs/FeCp_2_ sample with a 1:3 weight ratio (luminous power = 210 mW).

Results related to ignition thresholds, power values referring to the illumination area of 0.64 cm^2^ (square spot 0.8 cm × 0.8 cm as in the previous test), and power density obtained for IG-MWCNTs/FeCp_2_ with weight ratios from 1:4 to 5:1 are reported in Table 3. In addition, the related OD of used filters, besides ignition delays Δt, are indicated. The table also reports the comparison between ignition thresholds obtained for the two nanoparticle typologies (IG and HP MWCNTs).

For 5:1, 4:1, 3:1, and 2:1 weight ratios, sample ignition was obtained with a time delay ∆*t* higher than 2 s and with a low combustion intensity; for these reasons, the power corresponding to the neutral optical filter used was considered the ignition threshold. For 1:1, 1:2, 1:3, and 1:4 weight ratios, the ignition threshold values were not directly obtainable; in these cases, using a neutral optical filter with OD equal to 0.2, sample ignition was readily observed. On the contrary, no ignition was observed when using filter with OD of 0.3. Since the luminous power related to a filter with OD of 0.2 was 210 mW and that relative to a filter with OD of 0.3 was 180 mW, for these samples, as reported in the table footnote, an ignition threshold value of 190 mW was extrapolated. 

By comparing the ignition thresholds found for the two CNT typologies, as expected because of the slight difference between the dimensional features of the two CNT typologies, a substantial agreement between the obtained values was observed for all considered weight ratios. Therefore, previous considerations regarding a substantial reduction (between 20% and 40%) of ignition thresholds obtained with a LED source, with respect to the CW Xe lamp, as well as the trend of the thresholds as a function of ferrocene percentage in the mixture, were also confirmed for IG-MWCNTs. Furthermore, the obtained results establish that the IG-MWCNTs typology is a viable alternative to the HP-MWCNTs typology, with the cost of the industrial-grade typology being considerably lower than that of the high-purity typology. Table 4 reports the calculated minimum ignition energies (MIE) referring to an illumination area equal to 0.64 cm^2^ and the relative energy density, varying the weight ratio of IG-MWCNTs/FeCp_2_ samples; these values were calculated by considering the luminous power emitted by LED source without a neutral optical filter (340 mW) and the ignition delay of samples, extrapolated from the acquired video.

### 3.2. Results of Ignition Tests of IG-MWCNTs/FeCp_2_ Using Pulsed LED Source

**Figure 9:**
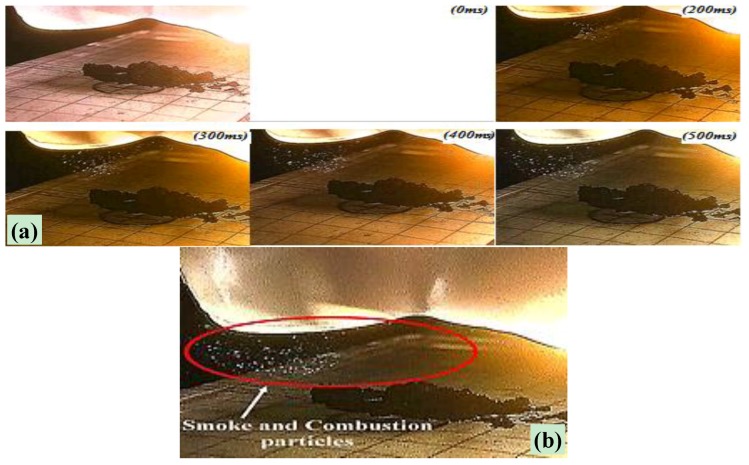
Frame sequence relative to photo-ignition test of IG-MWCNTs/ferrocene sample with 3:1 weight ratio and light pulse duration equal to 160 ms (**a**); view of the emitted smoke and particles soon after light pulse (**b**).

Ignition tests of dry IG-MWCNTs/FeCp_2_ samples, by means of a single luminous pulse, were carried out using the experimental setup shown in Figure 2, with the LED source composed of four XHP70 LEDs. As previously mentioned in the experimental section, for obtaining the MIE values, the luminous pulse energy was changed by acting on time duration of the light pulse. Therefore, starting with a long pulse duration, once we observed sample ignition we substituted the sample each time with a new one with the same weight ratio; the duration of the light pulse was progressively reduced until no combustion occurred. Figure 9a reports frames captured during the ignition test of an IG-MWCNTs/FeCp_2_ sample with a weight ratio of 3:1 and a light pulse duration of 160 ms. By observing the frames soon after the light pulse at 0 ms (those captured from 200 ms onwards), the emitted smoke and burnt particles plume are clearly visible, as highlighted in Figure 9b. Therefore, by determining the minimum pulse duration for obtaining sample ignition, MIE values were calculated. 

As an example, for the mixture with a 4:1 weight ratio, the minimum pulse duration for ignition occurrence was 110 ms. Since the emitted power (*P*) from the LED source is equal to 10.15 W, and assuming constant luminous power during the whole light pulse, the MIE value, referring to the effective illuminating area (estimated as 4.2 cm^2^ in the previously reported discussion), was calculated according to the following formula:

(5) MIE=P·Δt=10.15 W×110 ms=1.12 J

Therefore, the MIE value referring to the unit area is equal to 266 mJ/cm^2^, calculated as follows:

(6) $$MIE\;\left[\frac{\mathrm{mJ}}{{\mathrm{cm}}^{2}}\right]=\frac{MIE\left[J\right]}{A_{eff}}=\frac{1.12\;J}{4.2\;{\mathrm{cm}}^{2}}=266\;\frac{\mathrm{mJ}}{{\mathrm{cm}}^{2}}$$

**Figure 10:**
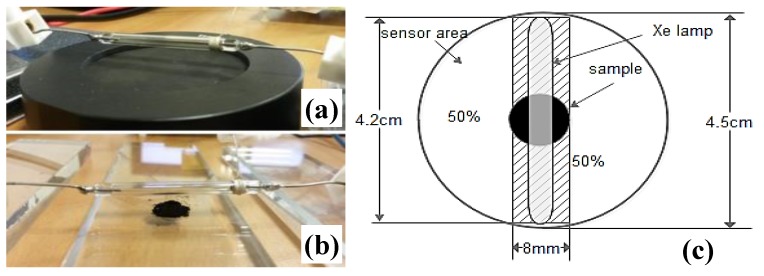
L4040 Xe lamp placed at a distance of 4 mm from the pyroelectric sensor surface (**a**); sensor substituted with sample preserving the same distance (**b**); and hypothesized energy spatial distribution, with sample placed along the lamp axis (**c**).

The MIE values obtained by using a pulsed LED source, compared with those obtained by means of the Xe lamp reported in our previous works [14,15,16,17], are shown in Table 5. As reported in [14,15,16,17], using a 50J Xe lamp (Figure 10a) placed at a distance of 4 mm from the samples (Figure 10b), MIE values were determined for each considered concentration by weight of samples. For measuring the luminous energy emitted from a pulsed Xe lamp, an energy/power meter (THORLABS PM100D, Newton, NJ, USA) equipped with a pyro-electric sensor (THORLABS ES100C) was used to determine the luminous energy hitting the whole sensor-sensitive area (15.90 cm^2^). Therefore, in following table, MIE values are reported assuming a uniform energy distribution on the whole sensor area (indicated with *a* apex).

However, since samples were placed just below the Xe lamp at its midpoint, as shown in Figure 10b, for taking into account a correct energy spatial distribution and considering only the energy density of the sample, it was assumed that only 50% of the total energy on the sensor area was contained in the rectangular surface centered along the lamp axis, as highlighted in Figure 10c, with dimensions of 42 mm length (Xe lamp length) and 8 mm width. The remainder (50%) of the total energy was uniformly distributed on the remaining area (Figure 10c). Therefore, the energy density interacting with MWCNTs/FeCp_2_ samples was calculated by considering only 50% of the measured energy and dividing by the rectangular area ($S_{1}=4.2\;\mathrm{cm}\;\times \;0.8\;\mathrm{cm}=3.36\;{\mathrm{cm}}^{2}).$ These energy densities are reported in the following table (Table 5), indicated with the *b* apex.

As is evident from Table 5, the MIE values trend is very similar for both the light source typologies used; this demonstrates the consistency of the obtained results, in fact, as reported in [16,17]: samples with a higher amount of MWCNTs than the metallic catalyzer (i.e., ferrocene) have a lower ignition threshold. This result contradicts other studies reported in the literature; in particular, in [10,11,12,13], samples with a higher amount of iron nanoparticles have a lower ignition threshold. However, in these studies a different CNT typology (i.e., SWCNTs) and a different metallic catalyzer were used.

Comparing the MIE values obtained using both luminous source typologies, those related to the LED source are considerably higher than those related to the Xe lamp (with a percentage difference between 106% and 166%). This difference is due to the different power intensities provided by the two source typologies, because of their different transients related to the emitted light power; in fact, for the LED source, with a constant absorbed current, the emitted luminous intensity is stable but considerably lower than the luminous power emitted by the pulsed Xe lamp (soon after the turning on of the Xe-lamp). For this reason, relative to the LED source used, light pulse durations between 100 ms and 200 ms were needed for inducing the MWCNTs/FeCp_2_ samples’ ignition. 

The dependence of MIE values on the duration of light pulses, relative to SWCNT samples with 50% Fe nanoparticle photo-ignition and the use of a Xe lamp, was analyzed in [10,11,12,13]. The results demonstrate that the speed with which a certain amount of energy is provided to a sample plays an important role; in particular, a lower amount of light energy is needed for triggering the combustion of a sample when shorter luminous pulses, but with higher luminous intensity, are used.

The lower luminous intensity of the LED source justifies the higher MIE values obtained with respect to those obtained with an Xe lamp, as discussed above, and, consequently, also the higher duration of light pulses for triggering sample ignition.

**Figure 11:**
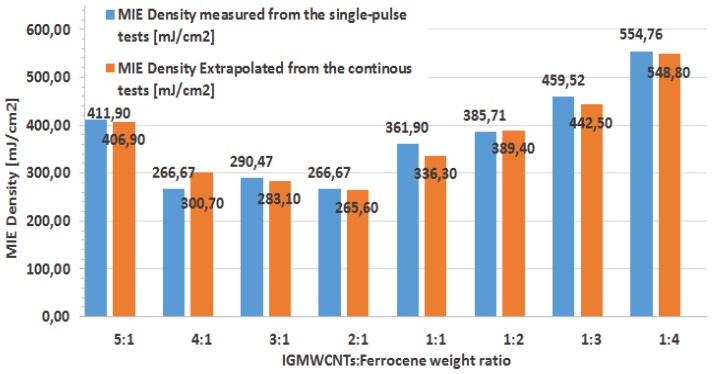
Comparison of MIE values obtained using LED sources from the single-pulse tests and extrapolated by estimating the minimum ignition delays of IG-MWCNTs/FeCp_2_ samples from continuous-emission tests.

Finally, a comparison between MIE values obtained from ignition tests of dry IG-MWCNTs/FeCp_2_ samples using a single luminous pulse and those related to a continuous-emission LED source (previously shown in Table 4) is given in Figure 11.

Values related to the continuous-emission tests were obtained, as previously mentioned, by extrapolating, from acquired videos, the ignition delay of the samples given a known luminous intensity emitted from the LED source (340 mW). As is visible in Figure 11, the obtained trend of MIE values related to single luminous pulse tests is in excellent agreement with that derived from tests using the continuous-emission LED source, with the maximum deviation within about ±10%. This deviation could be due to the erroneous identification (visual) of the precise starting instant of combustion process and to uncertainty about the instant of acquisition (with the frames being temporally spaced by 33.3 ms).

## 4. Discussion

The functionalized CNTs show improved properties, allowing easier synthesis of novel nanomaterials and nanostructures [28,29]. Specifically, CNT functionalization through porphyrin allowed for obtaining remarkable results, making these nanomaterials suitable for innovative applications [30]. In the following, results of photo-ignition tests carried out with IG-MWCNTs/FeCp_2_ samples enriched with porphyrin are reported.

### 4.1. Photo-Ignition Mechanism of IG-MWCNTs/FeCp_2_ Samples Enriched with Porphyrin

The functionalization of the CNTs surface is a powerful means for enhancing nanotube solubilization in a proper solvent, allowing an easier and extended characterization but also a better material reactivity [31]. In this context, the synthesis of nanohybrid materials, given by conjunction of aromatic polycyclic molecules (as porphyrin) and conjugated polymers, was obtained by π–π interaction. In particular, the implementation of photo-sensitized nanohybrid mono-chromophores (based on SWCNTs) for photovoltaic applications, thanks to their excellent light harvesting and charge transport properties, was demonstrated in [26]; these nanohybrid materials are based on SWCNTs and donor molecules. As demonstrated by the authors in [26], some composites containing porphyrin derivatives allow for rapid charge separation and slow recombination, key factors for obtaining high light conversion efficiencies.

A few years ago, it was demonstrated that the presence of porphyrins impregnated onto a semiconductor surface improved the photo-induced charge separation promoted by UV irradiation [25]. The positive holes readily delocalized in the macrocyclic structure of the porphyrins increased the lifetime of the photo-produced pairs; consequently, the slower recombination rate produced the highest amount of hole–electron pairs available to set off the electron transfer reactions. 

Moreover, the immobilization of tetra-phenyl-porphyrins (free-base H_2_P and zinc complex ZnP) on SWCNTs surface, resulting in a structure used as electron donor–acceptor, was obtained in [32], where the electronic interaction between the SWCNTs’ surface and porphyrins (H_2_P and ZnP), through π-orbitals of the two molecules, was demonstrated. In addition, in [33], a study on the bond between π-orbitals of perinil–porphyrin and SWCNTs was carried out; absorption transients of the two molecules show a rapid charge transfer (with time constant of about 260 fs) with a rapid charge recombination on a picosecond time scale. 

The first reason for using porphyrins concerns the improvement of absorption features of the MWCNTs/FeCp_2_ compound [34]; a consequence of the strong conjugation of these molecules (of porphyrin) is their intense absorption bands in the visible region, resulting in a higher concentration of reactive radical species (FeCp_2_^+^, MWCNTs^−^) that can react with oxygen, triggering ignition.

The second reason for using porphyrins is related to the properties of photoexcitation and charge transfer of such molecules; the lifetime of their singlet state is estimated at 1–10 ns for free-base porphyrin, i.e., without metal ions in its cavity, and about 1 ns for meso-substituted porphyrin (with metal ions in its cavity), whereas the lifetime of their triplet states is longer than 400 µs. According to the chemical–physical interpretation of the MWCNTs/FeCp_2_ photo-ignition reported in [6], the photo-ignition phenomenon is due to the charge transfer between FeCp_2_ and MWCNT molecules, generating radical species (FeCp_2_^+^, MWCNTs^−^) that are highly reactive; this charge transfer requires a time constant 10–100 times lower than the lifetimes of the electronic states of the two molecules. Therefore, photo-induced electronic transfer between FeCp_2_ and MWCNTs molecules is possible only for the triplet states, which have the required long lifetimes [35]. Hence, by adding those molecules that have a long lifetime, the probability that photo-induced charge transfer between porphyrin and MWCNTs will take place increases; consequently, a higher concentration of radical species generated by photoabsorption and thus higher photo-reactivity of sample is obtained.

**Figure 12:**
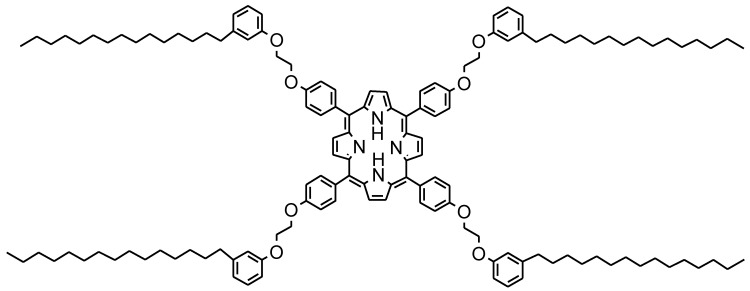
Molecular structure of the porphyrin H_2_Pp used in this work.

The molecular structure of the lipophilic porphyrin H_2_Pp employed in this work is shown in Figure 12. H_2_Pp consists of lipophilic chains based on cardanol (3-(pentadeca-8-enyl)-phenol) and was prepared for the first time as reported in [27]. 

Thanks to its processability, many environmentally friendly processes were based on this peculiar molecule based on cardanol [36,37,38,39,40]. 

H_2_Pp can be characterized by spectroscopic analysis, with the absorption spectrum of the porphyrin considered in different conditions [40]. 

As reported in Table 6, the resulting UV–Vis spectrum for H_2_Pp powder exhibits strong absorption in two spectral regions, known as the Soret band or B-band (~380–420 nm) and Q-bands (500–800 nm), respectively. These spectral components are very close to the two dominant spectral components of the XHP70 white power LED used (at 420 nm and 560 nm, as previously shown in Figure 3). In this way, a higher absorption efficiency is obtained for the compounds enriched with H_2_Pp with respect to that obtained using compounds without porphyrin, allowing a higher generation of free electron–hole pairs, and so resulting in a greater production of reactive radical species that can trigger the combustion process.

### 4.2. Sample Preparation of MWCNTs/FeCp_2_ Mixture Enriched with Porphyrin

Samples of IG-MWCNTs/FeCp_2_ enriched with porphyrin H_2_Pp were prepared by adding the desired amount of porphyrin powder to the pre-mixed compounds (IG-MWCNTs/FeCp_2_), each of weight 20 mg and different weight ratios (from 5:1 to 1:4). The resulting mixture, placed in a ceramic mortar, was crushed to obtain a homogenous compound without agglomerations. In this first experimental phase, we decided to add 3 mg of porphyrin to the premixed compounds (IG-MWCNTs/FeCp_2_), regardless of the sample weight ratio considered, resulting in a total weight for each mixture of 23 mg.

Hence, three different samples (each of about 7.6 mg) were obtained from each mixture weight ratio; in this way, three distinct ignition tests were performed on each compound.

In addition, for verifying the relationship between ignition thresholds and the amount of porphyrin powder used in the mixture, further samples of IG-MWCNTs/FeCp_2_ with a 2:1 weight ratio (this concentration was chosen because it had the lowest ignition thresholds in the single-pulse tests) were prepared by adding 8 mg of porphyrin to 20 mg of compound, thereby obtaining mixtures of 28 mg. From each compound, and also in this case, three different samples were obtained (each 9.3 mg) for carrying out three distinct ignition tests. Table 7 reports the composition of samples enriched with porphyrin, prepared for the photo-ignition tests.

### 4.3. Ignition Tests of IG-MWCNTs/FeCp_2_ Mixtures Enriched with Porphyrin 

Since the results related to MIE values (reported in Figure 11) obtained using continuous-emission and pulsed LED source show slight/minimum differences between them, for verifying the effects of porphyrin added to IG-MWCNTs/FeCp_2_ samples, the minimum ignition energy was determined for each prepared mixture weight ratio by employing the experimental setup based on the continuous-emission LED source shown in Figure 1. MIE values were determined by extrapolating, from acquired videos, the ignition delay of the tested sample and measuring the luminous intensity emitted from the LED. Obtained MIE densities for igniting the samples enriched with H_2_Pp are shown in Table 8; these values are also compared in Table 8 with those obtained with samples without porphyrin.

By comparing the MIE values obtained for IG-MWCNTs/FeCp_2_ samples enriched or not with porphyrin, a considerable reduction (on average of 25%), for all considered weight ratios, is evident. This is due to the better features of charge separation and recombination of samples enriched with porphyrin. Therefore, the obtained results confirm that the use of porphyrin improves the photo-reactivity of nanomaterials impregnated with it, as reported in more detail in [34], where the porphyrin properties were exploited in other processes for different aims—in particular, for investigating the photodegradation of 4-nitrophenol in aqueous suspension.

**Figure 13:**
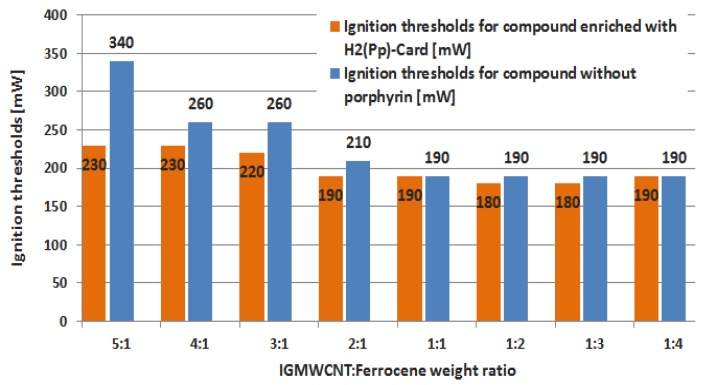
Comparison between the extrapolated ignition thresholds of dry IG-MWCNTs/FeCp_2_ samples enriched with H_2_Pp porphyrin (orange histogram) and those obtained for samples without porphyrin (blue histogram).

Besides MIE values, the minimum luminous power needed for igniting samples enriched with porphyrin were also determined; as previously discussed, the resolution on the luminous power is limited by the available OD values of used filters; therefore, in some cases, ignition threshold values were extrapolated based on the experimental observations of the sample ignition process (i.e., ignition delay Δt and intensity of combustion process). The histogram of Figure 13 reports the extrapolated values of ignition thresholds for samples enriched with 3 mg of porphyrin, for each tested weight ratio, and also the comparison between these values with those derived for samples without porphyrin. Ignition threshold of IG-MWCNTs/FeCp_2_ sample with 2:1 weight ratio enriched with 8 mg of porphyrin was equal to 180 mW (not reported in the histogram); therefore, by comparing this threshold value with that related to the sample with the same weight ratio but 3 mg of porphyrin (190 mW), the difference is not significant.

Comparing the minimum ignition power of dry IG-MWCNTs/FeCp_2_ samples enriched or not with H_2_Pp porphyrin, a slight reduction in the thresholds for samples enriched with porphyrin was observed. The reduction is more evident in terms of energies needed for obtaining the ignition (as reported previously in Table 8); we suppose that this is due to the greater yield of radical species, highly reactive, of samples with porphyrin, resulting in a faster sample ignition (lower ignition delay).

**Figure 14:**
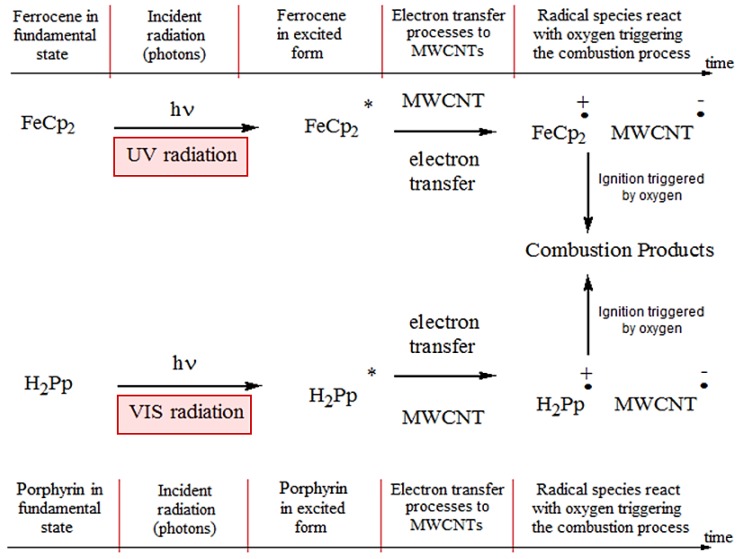
Schematic representation of the reaction occurring when the MWCNTs/FeCp_2_ mixture with added porphyrin absorbs the photons emitted from the used luminous source; H_2_Pp allows us to improve the VIS radiation absorption, thus increasing free hole-electron pairs by means of faster transfer and longer recombination time and so enhancing the combustion process.

We suppose that by adding the porphyrin powder to MWCNTs/FeCp_2_ samples, a parallel reaction mechanism occurs in the MWCNTs/FeCp_2_ photo-ignition process; as shown in Figure 14, related to the FeCp_2_ photoexcitation, the intermolecular electron transfer results in MWCNT^−^–FeCp_2_⁺ radical species, depending on the selected wavelength range of the incident radiation [6]. The photo-induced radical species, being highly reactive, can easily react with oxygen (O_2_), giving rise to a combustion reaction. 

The parallel reaction mechanism of Figure 14, related to H_2_Pp photoexcitation, gives rise to a MWCNT^−^–H_2_Pp⁺ charge-separated state, as expected by the photo-induced intermolecular electron transfer in the hybrid nanomaterial used. Unlike with the MWCNTs/FeCp_2_, intermolecular electron transfer, where the photo-induced process of charge separation is mainly promoted by UV light components, the MWCNTs/H_2_Pp intermolecular electron transfer is mainly ascribed to the VIS light components of the radiation source. In this way, the spectral components of the used LED source (corresponding to 420 nm and 560 nm) contribute significantly to the formation of the radical species (MWCNT^−^–H_2_Pp⁺). 

## 5. Conclusions

In this research work, the ignition mechanism of MWCNTs/FeCp_2_ samples, photo-ignited by means of different LED sources (continuous-emission and pulsed), is analyzed. To our knowledge, this is the first demonstration of the MWCNTs/FeCp_2_ photo-ignition with this kind of radiation source. By using a continuous-emission LED, ignition tests on dry MWCNTs/FeCp_2_ samples were carried out by employing two different typologies of MWCNTs (HP-MWCNTs and IG-MWCNTs) with different dimensional and purity features. The obtained minimum luminous power values needed for igniting the samples for each considered weight ratio are similar for both MWCNTs typologies, showing a similar trend of ignition thresholds; in particular, a higher amount of metallic catalyzer in the sample results in a lower ignition threshold. By comparing the obtained results with those related to the use of a CW Xe lamp, as reported in our previously published research work [6], it is evident that there was a reduction in ignition thresholds, from 20% up to 40%, for all the tested weight ratios.

Furthermore, for calculating the minimum ignition energies, ignition delays were extrapolated from the high-definition videos acquired during the tests carried out for each ignited sample. These energy values were then compared, showing an optimal agreement, with those obtained from the single-pulse experimental tests, performed by employing IG-MWCNTs/FeCp_2_ samples and an LED source driven and controlled by a specially designed electronic unit. These MIE values, also compared with those obtained in our previous research work employing a pulsed Xe lamp [14,15,16,17], show a trend similar to the function of sample weight ratios, but are higher because of the lower luminous intensity emitted from the LED source than the Xe lamp. For obtaining a higher luminous intensity from LED, further improvements concerning the optics used for collimating the light beam emitted from LEDs have to be performed. In fact, the solution adopted in this research work undergoes huge luminous intensity leakages (about 70% with respect to the luminous intensity emitted from “naked” LED) due to the high F-Number, namely the ratio between lens’ focal length and diameter (because of the small dimensions of used lenses). Therefore, a trade-off is needed between the high energy densities, obtained by collimating the emitted light as much as possible, and the luminous intensity leakages [41].

Finally, improvements in the ignition mechanism were obtained by adding porphyrin molecules to the IG-MWCNTs/FeCp_2_ samples. The ignition tests carried out on samples enriched with H_2_Pp porphyrin demonstrated a significant reduction of MIE values, between 10% and 40%, compared to those obtained for samples without porphyrin, for all the considered weight ratios.

## Figures and Tables

**Table 1:** Prepared MWCNTs/FeCp_2_ weight ratios and ferrocene percentage by weight.

Weight Ratio of the Sample		Ferrocene Percentage by Weight
MWCNTs	Ferrocene	
5	1	≈16.7%
4	1	20%
3	1	25%
2	1	≈33%
1	1	50%
1	2	≈67%
1	3	75%
1	4	80%

**Table 2:** Comparison between ignition thresholds obtained using the continuous-emission LED source and the continuous-wave (CW) Xenon lamp; percentage differences are also reported.

Weight Ratios		LED Source		XENON Lamp		Percentage Difference (%)
HPMWCNTs	Ferrocene	Luminous Power (Illumination Area 0.64 cm^2^) (mW)	Luminous Power Density (mW/cm^2^)	Luminous Power (Illum. Area 0.634cm^2^) (mW)	Luminous Power Density (mW/cm^2^)	
4	1	260 (OD = 0.1 filter)	406	435	684	−41
3	1	240 ^1^ (OD = 0.1 filter)	375	360	566	−34
2	1	210 (OD = 0.2 filter)	328	290	456	−28
1	1	210 (OD = 0.2 filter)	328	275	432	−24
1	2	210 (OD = 0.2 filter)	328	250	393	−17
1	3	170 (OD = 0.1 + 0.2 filters)	266	240	377	−30
1	4	180 (OD = 0.3 filter)	281	270	425	−33

^1^ This value was extrapolated based on experimental observation related to the estimated ignition delay and ignition intensity visually evaluated from the acquired high-resolution videos.

**Table 3:** Comparison between ignition thresholds obtained for IG-MWCNTs/ferrocene and HP-MWCNTs/ferrocene samples, as a function of weight ratios.

Weight Ratios		LED Source		XENON Lamp		Percentage Difference (%)
HPMWCNTs	Ferrocene	Luminous Power (Illumination Area 0.64 cm^2^) (mW)	Luminous Power Density (mW/cm^2^)	Luminous Power (Illum. Area 0.634 cm^2^) (mW)	Luminous Power Density (mW/cm^2^)	
5	1	340 (without neutral filter)	531	-	-	-
4	1	260 (OD = 0.1 filter)	406	260	406	0
3	1	260 (OD = 0.1 filter)	406	240	375	+8
2	1	210 (OD = 0.1 filter)	328	210	328	0
1	1	190 (OD = 0.2 filter) ^1^	297	210	328	−10
1	2	190 (OD = 0.2, ∆*t* = 933 ms)^1^	297	210	328	−10
1	3	190 (OD = 0.2, ∆*t* = 1.166 s) ^1^	297	170	266	+10
1	4	190 (OD = 0.2, ∆*t* = 1.133 s) ^1^	297	180	281	+5

^1^ Measurements extrapolated from experimental observations.

**Table 4:** Calculated MIE values for different IG-MWCNTs/ferrocene weight ratios.

Weight Ratios		Energy (Illumination Area 0.64 cm^2^) (mJ)	Energy Density (mJ/cm^2^)
IG-MWCNTs	Ferrocene		
5	1	260.4	406.9
4	1	192.4	300.7
3	1	181.2	283.1
2	1	170.0	265.6
1	1	215.2	336.3
1	2	249.2	389.4
1	3	283.2	442.5
1	4	351.2	548.8

**Table 5:** Comparison of MIE density values obtained using pulsed LED source and pulsed Xenon lamp; for the Xe lamp, MIE densities were estimated by considering a uniform energy distribution (1 apex) and energy referred to the rectangular area (2 apex). Moreover, percentage differences between MIE densities obtained with LED source and Xe lamp (rectangular area, 2 apex) are reported.

HPMWCNTs	Ferrocene	LED Source		XENON Lamp	Percentage Difference (%)
		Minimum Pulse Duration (ms)	Energy Density (mJ/cm^2^)	Energy Density (mJ/cm^2^)	
5	1	170	411.9	-	-
4	1	110	266.67	51.36 ^1^–121.49 ^2^	+119
3	1	120	290.47	58.75 ^1^–139.00 ^2^	+109
2	1	110	266.67	54.63 ^1^–129.25 ^2^	+106
1	1	150	361.90	64.83 ^1^–153.39 ^2^	+135
1	2	160	385.71	69.74 ^1^–165.00 ^2^	+133%
1	3	190	459.52	88.75 ^1^– 209.99 ^2^	+119%
1	3	240	554.76	87.96 ^1^–208.11 ^2^	+166%

^1^ Considering the whole sensor area. ^2^ Refers to the estimated rectangular area.

**Table 6:** UV–Vis data of H_2_Pp obtained in different conditions and with different spectroscopic techniques (UV–Vis spectrum data and DRS (diffuse reflectometry spectroscopy) spectrum) and comparison with the LED emission spectrum peaks.

Measure Condition ^1^	Absorption λ_max_/nm (Soret and Q Bands) ^1^	Emission Spectrum Peaks
	H_2_Pp	CREE XHP70 LED
Solution in CH_2_Cl_2_	421, 515, 555, 593, 649	-
DRS in powder	396, 519, 558, 596, 651	420–560
DRS on semiconductor	440, 523, 562, 609, 652	-

^1^ As reported in the supplementary information of [40].

**Table 7:** Twenty mg of IG-MWCNTs/FeCp_2_ compounds enriched with 3 mg and 8 mg (last row) of porphyrin and the percentages of porphyrin in each sample.

Weight Ratio ^1^	IG-MWCNTs (mg)	FeCp_2_ (mg)	Porphyrin	
			(mg)	(%)
5:1	16.67	3.33	3	13.0
4:1	16	4	3	13.0
3:1	15	5	3	13.0
2:1	13.33	6.67	3	13.0
1:1	10	10	3	13.0
1:2	6.67	13.33	3	13.0
1:3	5	15	3	13.0
1:4	4	16	3	13.0
2:1	13.33	6.67	8	28.6

^1^ Weight ratio of IGMWCNTs/FeCp_2_ compound.

**Table 8:** Comparison between MIE densities obtained for IG-MWCNTs/FeCp_2_ samples both enriched and not with H_2_Pp porphyrin and related percentage differences of energy reduction.

IGMWCNTs	FeCp_2_	Without Porphyrin	With Porphyrin	Percentage Difference (%)
		Energy (mJ/cm^2^)	Energy (mJ/cm^2^)	
5	1	406.9	351.8	−13
4	1	338.4	270.5	−20
3	1	297.8	189.3	−36
2	1	284.1	152.9	−46
1	1	306.1	262.5	−14
1	2	382.5	356.1	−7
1	3	436.4	346.8	−20
1	4	546.6	481.0	−12
